# The Clinical Laboratory: Alimentary Diseases

**Published:** 1913-10-25

**Authors:** 


### THE CLINICAL LABORATORY.

### I.—Alimentary Diseases.

The investigation of cases of disease of the alimentary canal requires a well-equipped clinical laboratory. In the diagnosis of some cases one must take into consideration not only the ordinary clinical examination but also the data obtained in the laboratory from the examination of the fæces, stomach contents, urine, and blood, together with the results of an examination by the $x$-rays.

The writer usually commences the pathological examinations by a careful search for "occult" blood in the fæces, for the presence of "occult" blood points to some ulcerative lesion in the alimentary canal, whilst its absence is almost sufficient to exclude cancer of the stomach. If a simple ulcer is suspected six successive specimens of the fæces are examined before a negative result is accepted. Mucus and pus from the colon, if present in any amount, would be noticed during these examinations and would direct the course of further investigations.

After the search for "occult" blood has terminated (not before) the stomach contents are examined after a test breakfast. If there be any reason to suspect motor insufficiency a test dinner is given for the purpose of determining the motor powers of the stomach. Finally, in all cases where there are intestinal symptoms, especially diarrhœa, the patient takes Schmidt's test diet for three days and the fæces are examined by Schmidt's methods. When there is any reason to suspect disease of the pancreas or the biliary tract Cammidge's special tests for the urine and fæces are used at this stage. This appears to be a very tedious method of examination, but it is not so troublesome as might appear at first sight, for in reality each examination for "occult" blood takes about five minutes, the examination of the test breakfast about the same time (in a properly equipped laboratory), whilst Schmidt's method of examining the fæces, after a little practice, requires attention for about twenty minutes. Cammidge's tests alone require not only a considerable expenditure of time but also considerable manipulative skill. Whilst the different tests are being conducted the diet and general régime of the patient must be under careful control. That there are many loop-holes for error will appear from the details of the tests.

### "Occult" Blood.

The recognition of "occult" blood in the stools represents one of the great advances in diagnosis of gastric disease due to the energy of the German clinicians. It had long been known that blood shed into the stomach or intestines lost its red colour and become black (melæna), but Boas showed that blood might be detected in fæces of quite a normal appearance and colour and that the presence of such "occult" blood was of the greatest clinical significance. These observations have been amply confirmed, although there is not yet absolute agreement as to the best method of detecting blood nor upon the exact clinical value of its discovery in the fæces.

For the detection of the blood simple microscopic examination is quite useless, whilst spectroscopic examination is not sufficiently delicate, so that we are thrown back upon chemical methods. Numerous attempts have been made to obtain hæmin crystals, but none have had much success, and we must rely upon a series of reactions which depend on the property which hæmoglobin and some of its derivatives possess of liberating nascent oxygen from hydrogen peroxide, the nascent oxygen being detected by different colour reactions, the most useful of which are the guaiacum, benzidin, and phenolphthalein tests. As is well known all these reactions are open to fallacies in that other substances possess the same catalytic property of decomposing peroxide, but the conditions of the experiment can be so arranged that the results are quite reliable.

*Precautions.*—For at least three days before the tests are performed the patient must take a diet from which the following articles are excluded: meat and all foods prepared from meat, fish, fowl, green vegetables, and all drugs except a saline purge. Hæmorrhoids must be excluded. The motions must be kept soft by the administration of a saline purge every morning—otherwise the passage of hard masses of fæces with the accompanying strain is apt to abrade the anal mucosa and result in minute hæmorrhages which escape observation and yet are sufficient to give positive reactions.

*Technique of the Tests.*—First of all must be prepared an acid ethereal extract of the fæces. A piece the size of a small filbert is transferred to a large test tube and gently rubbed up by means of a glass rod with a few cubic centimetres of glacial acetic acid. About 20 c.c. of ether are now added and the whole shaken or stirred. The suspension is then filtered through a Swedish filter paper. The filtrate is used for both benzidin and guaiacum tests. Whilst the extract is filtering one prepares a tincture of guaiacum by shaking a knife point of the powdered resin with a little absolute alcohol. A benzidin solution is made by warming a little of the substance (Merck's "Benzidin zum Blutnachweis") in glacial acetic acid. One is now ready to perform the tests. To 2 c.c. of the ethereal extract add two or three drops of the guaiacum tincture and 2 c.c. of hydrogen-peroxide solution. The appearance of a blue colour is proof of the presence of blood. In some cases the change of colour is so indefinite, owing to the presence of other pigments, that a confirmatory test must be used, and for this purpose benzidin is very useful. The benzidin solution in glacial acetic acid is divided into two portions and to one of them is added a few drops of the acid ethereal extract, whilst the second serves as a (very necessary)

control. To both are added several cubic centimetres of hydrogen-peroxide solution. The control should become turbid, but should show no trace of blue within one minute. If there be a definite blue coloration, arising within one minute, in the tube containing the extract the reaction may be looked upon as positive. An extract which has given a very indefinite result with guaiacum will give a very definite result with benzidin. The phenolphthalein test is the most simple of all the tests. An emulsion of fæces is made by stirring up a small fragment of material with water and a few drops of this is added to 2 c.c. of Meyer's reagent followed by a single drop of hydrogen-peroxide solution. A change of colour to pink or red is an indication of the presence of blood. Meyer's reagent is prepared by boiling together two parts of phenolphthalein, twenty parts of caustic potash, and ten parts of zinc dust with 100 c.c. of distilled water. As the zinc dissolves in the alkali, liberating hydrogen, the brilliant red colour of the alkaline phenolphthalein fades and we obtain a colourless reduction product. The fluid is filtered and is ready for use. It keeps well for about a week, but if it becomes pink the colour can be discharged by boiling with a little zinc dust.

*The Utility of the Tests.*—The chief value of tests for "occult" blood lies in the fact that at a very early stage of a carcinoma of the stomach there is some loss of blood through the fæces, almost every stool giving a positive reaction. In simple ulcer there may also be some "occult" bleeding, but it is, as a rule, smaller in amount and ceases rapidly under treatment, for it is remarkable how rapidly in cases of simple ulcer the "occult" hæmorrhage clears up under rest in bed, combined with some suitable dietary, such as the Lenhartz treatment. In regard to ulcer, an important question, which has not yet been answered satisfactorily, is whether there is occult hæmorrhage which can be demonstrated by chemical methods in every case of duodenal or gastric ulcer. Dogmatic assertions have been made in either direction. The writer thinks that the position is about as follows. By using the guaiacum test as a criterion of the presence of blood one finds cases of ulcer (confirmed by operation) where there has been no "occult" blood in as many as six successive tests. When one places reliance on the benzidin test one finds many cases where the first two or three tests show a very feeble reaction. If the stool is submitted to a careful microscopical examination one usually finds some undigested muscle fibres, which must have been delayed somewhere in the alimentary canal. In some cases of this type subsequent operation has failed to demonstrate an ulcer. It appears that in some cases of ulcer the bleeding is so slight that it cannot be demonstrated by chemical tests unless these tests are so delicate that they are inapplicable under ordinary conditions. For instance, a positive benzidin test, with a negative guaiacum test, carries no weight unless the patient has taken a meat-free diet for the space of more than one week. The value of the benzidin test is that in cases where the guaiacum test is doubtful, owing to excessive pigment in the fæces obscuring the colour change, the former test—the benzidin—will enable a definite opinion to be given. It is to be noted that in cases of excessive vomiting, as in the pernicious vomiting of pregnancy, there may be hæmorrhage due to the straining; in cirrhosis of the liver there may be hæmorrhage due to congestion; in gall-stones there is occasionally some occult hæmorrhage: but these difficulties rarely arise. The test may be useful in revealing the presence of tuberculous ulceration as a complication of tuberculous peritonitis. It is said to give warning several days before manifest hæmorrhage in typhoid fever. The phenolphthalein test would appear to be specially useful in this connection, as it requires so little manipulation.

